# Amino Acids Activate Mammalian Target of Rapamycin (mTOR) Complex 1 without Changing Rag GTPase Guanyl Nucleotide Charging*

**DOI:** 10.1074/jbc.M113.528505

**Published:** 2013-12-11

**Authors:** Noriko Oshiro, Joseph Rapley, Joseph Avruch

**Affiliations:** From the Department of Molecular Biology and Diabetes Unit, Medical Services, Massachusetts General Hospital, Boston, Massachusetts 02114 and the Department of Medicine, Harvard Medical School, Boston, Massachusetts 02115

**Keywords:** Cell Signaling, GTPase, Insulin, Lysosomes, mTOR Complex (mTORC), Rag GTPase, Amino Acids, Raptor

## Abstract

Activation of mammalian target of rapamycin complex 1 (mTORC1) by amino acids is mediated in part by the Rag GTPases, which bind the raptor subunit of mTORC1 in an amino acid-stimulated manner and promote mTORC1 interaction with Rheb-GTP, the immediate activator. Here we examine whether the ability of amino acids to regulate mTORC1 binding to Rag and mTORC1 activation is due to the regulation of Rag guanyl nucleotide charging. Rag heterodimers *in vitro* exhibit a very rapid, spontaneous exchange of guanyl nucleotides and an inability to hydrolyze GTP. Mutation of the Rag P-loop corresponding to Ras^Ser-17^ abolishes guanyl nucleotide binding. Such a mutation in RagA or RagB inhibits, whereas in RagC or RagD it enhances, Rag heterodimer binding to mTORC1. The binding of wild-type and mutant Rag heterodimers to mTORC1 *in vitro* parallels that seen with transient expression, but binding to mTORC1 *in vitro* is entirely independent of Rag guanyl nucleotide charging. HeLa cells stably overexpressing wild-type or P-loop mutant RagC exhibit unaltered amino acid regulation of mTORC1. Despite amino acid-independent raptor binding to Rag, mTORC1 is inhibited by amino acid withdrawal as in parental cells. Rag heterodimers extracted from ^32^P-labeled whole cells, or just from the pool associated with the lysosomal membrane, exhibit constitutive [^32^P]GTP charging that is unaltered by amino acid withdrawal. Thus, amino acids promote mTORC1 activation without altering Rag GTP charging. Raptor binding to Rag, although necessary, is not sufficient for mTORC1 activation. Additional amino acid-dependent steps couple Rag-mTORC1 to Rheb-GTP.

## Teaser

**Background:** Signaling by mTOR complex 1 requires its amino acid-stimulated binding to Rag GTPase heterodimers.

**Results:** mTOR complex 1-Rag binding *in vitro* is independent of Rag guanyl nucleotide charging, and withdrawal of amino acids from cells does not alter Rag GTP charging.

**Conclusion:** Amino acids promote Rag binding to mTOR complex 1 without changing Rag GTP charging.

**Significance:** Amino acids promote Rag-mTORC1 binding by undefined mechanisms.

## Introduction

Mammalian target of rapamycin complex 1 (mTORC1),^2^ the hetero-oligomeric assembly of mTOR, raptor, and mLST8 (1–3), is a giant protein (Ser/Thr) kinase that serves as a major regulator of transcription, translation, and autophagy in eukaryotic cells (4). The activity of mTORC1 in mammalian cells is critically dependent on the small GTPase Rheb, which binds directly to the mTOR catalytic domain and, in its GTP-bound state, promotes mTORC1 catalytic activity (5). mTORC1 signaling is regulated in a reciprocal manner by insulin/mitogens and stress, whose inputs largely converge on the tuberous sclerosis complex (TSC), a complex of Hamartin/TSC1, Tuberin/TSC2, and TBC1D7 that is the GTPase-activating protein for Rheb (6–8). Elimination of TSC function increases Rheb GTP charging to over 90% and gives a constitutive activation of mTORC1 that is not increased further by insulin or mitogens (9). Signaling by mTORC1 is also regulated by extracellular amino acids. In cell culture, increased amino acids can activate mTORC1 fully, whereas withdrawal of amino acids from the medium reversibly abolishes mTORC1 signaling and renders it resistant to stimulation by insulin (10). Amino acid withdrawal also abolishes mTORC1 signaling in TSC null cells but does not diminish the Rheb GTP charging in wild-type or TSC null cells (9). Amino acid withdrawal does diminish the binding of recombinant Rheb to mTOR. However, overexpression of Rheb can restore mTORC1 signaling in amino acid-deprived cells (11). Taken together, these findings support the view that the inhibition of mTORC1 by amino acid withdrawal is, at least in part, due to some interference with the ability of endogenous Rheb-GTP to bind endogenous mTORC1 (11).

Signaling by mTORC1 is also dependent on the Rag GTPases (12, 13), a novel family of four small GTPases, A–D, whose amino-terminal GTPase domains are followed by long C-terminal extensions. The Rags exist as constitutive heterodimers, with RagA or B complexed with RagC or D (14). Depletion of either Rag heterodimer partner inhibits mTORC1 signaling, and overexpression of the Rag heterodimer can rescue mTORC1 signaling from inhibition by amino acid withdrawal (12, 13). Although the wild-type Rag heterodimer enables a weak rescue, a heterodimer containing RagB^Q99L^ (a switch II mutation corresponding to Ras^Q61L^ that promotes Ras occupancy by GTP (15)) can more effectively restore mTORC1 signaling, and the additional mutation of the RagC/D partner at the residue corresponding to Ras^Ser-17^ (the mutation of which effectively restricts Ras to GDP binding in cells (16)), although having modest (RagD) or very little (RagC) activating effect by itself, yields a substantial further increase in RagB^Q99L^-stimulated mTORC1 signaling. Rag heterodimers containing the RagA^Thr-21^/RagB^Thr-54^ mutants (which correspond to Ras^Ser-17^) behave as dominant inhibitors, suppressing mTORC1 activity in amino acid-replete cells (12, 13). These findings led to the conclusion that activation of mTORC1 by the Rag heterodimer is achieved by enhanced GTP charging of the RagA/B partner, with a secondary assist by conversion of the RagC/D partner to the GDP-bound state. Notably, overexpression of the optimal Rag heterodimer in amino acid-deprived cells yields mTORC1 activation that is far short of that enabled by Rheb overexpression. Moreover, RagA^T21N^ does not inhibit the ability of overexpressed Rheb to restore mTORC1 signaling in amino acid-deprived cells (13). In *Drosophila*, dRheb is required for the growth-promoting effects of the activated dRag^Q61L^, whereas dRag is not required for the growth-promoting effects of dRheb (13). These results indicated that the Rag heterodimer acts upstream of or parallel to Rheb-GTP to activate mTORC1.

In fact, Sancak *et al.* (12) demonstrated that the RagA/B-RagC/D heterodimer associates directly with mTORC1 through an amino acid-stimulated binding to raptor. The binding of endogenous mTORC1 to the recombinant Rag heterodimer is much more avid than to recombinant Rheb. They reported that, as compared with wild-type Rag heterodimers, a Rag heterodimer containing RagB^Q99L^ exhibits greatly increased binding to raptor, whereas a Rag heterodimer containing a RagC or D mutant in the residue corresponding to Ras^Ser-17^ shows slightly increased raptor binding. Moreover, heterodimers containing a RagB^T54L^ mutant (corresponding to Ras^Ser-17^) exhibit greatly reduced raptor binding. These findings, which appeared to parallel the functional effects of the Rag mutants, led to the conclusion that the RagA/B partner, when GTP-bound, plays the primary role in raptor binding by the Rag heterodimer as well as in mTORC1 activation.

In amino acid-deprived HEK293T cells, mTOR is distributed diffusely in a punctate fashion throughout the cytoplasm. Readdition of amino acids is accompanied by a Rag- and raptor-dependent relocalization of mTOR to a perinuclear compartment that contains Rab7 and is enriched in recombinant Rheb. Cells expressing RagB^Q99L^ show a persistent perinuclear localization of mTOR despite amino acid withdrawal. The subcellular localization of the Rag polypeptides is not altered by amino acid withdrawal (12). Rags reside in this late endosomal/lysosomal compartment through a noncovalent association with the Ragulator complex, an assembly of five polypeptides anchored to the membrane by the myristoylated/palmitoylated protein p18/LAMTOR1 (17, 18). These results support the conclusion that amino acids, presumably by their ability to promote the binding of raptor to the Rag heterodimer, translocate mTORC1 into the membrane compartment containing Rheb, the proximate activator of mTORC1. This formulation provides a satisfying explanation for the Rheb requirement for Rag-stimulated mTORC1 signaling.

The conclusion that amino acids regulate the Rag binding to raptor *in vivo* by altering Rag guanyl nucleotide binding is primarily on the basis of the behavior of Rag mutants whose nucleotide binding was inferred from the effects of homologous mutations of Ras on its ability to bind and hydrolyze guanyl nucleotides. To examine the validity of this view, we characterized the ability of wild-type and mutant Rag GTPases to bind raptor/mTORC1 during transient expression in comparison with their ability to bind mTORC1 *in vitro* as a function of guanyl nucleotide charging. In addition, we examined the ability of wild-type and mutant Rag heterodimers to bind and hydrolyze guanyl nucleotides *in vitro* and the extent of Rag GTP charging in amino acid-replete and amino acid-deprived cells. We found that Rag mutations alter Rag heterodimer binding to mTORC1 in a similar way in transfection and *in vitro* but that Rag binding to mTORC1 *in vitro* is independent of Rag guanyl nucleotide charging. Altered mTORC1 binding of Rag mutants is attributable to structural effects of the mutations rather than altered Rag guanyl nucleotide charging. *In vitro*, Rag heterodimers show rapid binding and release of guanyl nucleotides, with little or no hydrolysis of GTP. In cells, Rag A/B is constitutively GTP-charged, whereas RagC/D is predominantly GDP-charged. Nevertheless, amino acid withdrawal, despite inhibiting mTORC1, does not alter Rag guanyl nucleotide charging. Thus, the mechanism by which amino acids promote the Rag-raptor interaction is independent of amino acid-induced changes in Rag guanyl nucleotide charging. Moreover, although overexpression of wild-type Rag heterodimers enables raptor binding in amino acid-deprived cells, Rag overexpression is insufficient to cause mTORC1 activation. Even heterodimers containing RagC^S75L^, which have enhanced binding to raptor, cause little mTORC1 activation unless partnered with RagA^Q66L^/RagB^Q99L^ switch II mutants. Thus, in addition to raptor binding, other productive interactions of the RagA/B GTPase domain are required to bring mTORC1 in approximation to Rheb-GTP for activation. Given the constitutively high RagA/B GTP charging, amino acid sufficiency must, as with raptor binding, also promote these other RagA/B interactions independently of altered Rag GTP charging.

## EXPERIMENTAL PROCEDURES

### 

#### 

##### Antibodies

Antibodies were obtained from the following sources. Anti-β-actin (catalog no. ab8227) and anti-LAMP2 (catalog no. ab25631) were from Abcam. mTOR (7C10) rabbit mAb (catalog no. 2983), raptor (24C12) rabbit mAb (catalog no. 2280), RagA/B (D8B5) rabbit mAb (catalog no. 4357), RagC antibody (catalog no. 3360), S6K [T389P], (1A5) mouse mAb (catalog no. 9206), 4EBP1 phospho-4E-BP1 (Thr-37/46) antibody (catalog no. 9459), GFP antibody (catalog no. 2555), and Myc tag (9B11) mouse mAb (catalog no. 2276) were from Cell Signaling Technology. Anti-HA.11 MC Ab, clone 16B12, and MMS-101P were from Covance. Anti-mTOR (N5D11) mouse IgG MC Ab (catalog no. 10343) and anti-human raptor (R984) rabbit IgG (catalog no. 28011) were from Immuno-Biological Laboratories. The ProteoExtract S-PEK antibody control kit, anti-Hsp90a mouse mAb (catalog no. EMD-17D7), anti-calnexin mouse mAb (catalog no. TO-5), anti-PARP-1 (Ab-2) mouse mAb (catalog no. C-2-10), and anti-vimentin/LN6 (Ab-1) mouse mAb (catalog no. V-9) were from Novagen. Anti-phospho-PRAS40 (Thr-246) (catalog no. 07-888) was from Millipore. Normal rabbit IgG (catalog no. sc-2027) and GST antibody (Z-5) (catalog no. sc-459) were from Santa Cruz Biotechnology. Anti-FLAG M2 was from Sigma-Aldrich. Rabbit PC antibodies against RagC were generated by immunization with the synthetic peptides CysETPLAGSYGAADamide (for immunoblot analysis and cytochemistry) and CysGHQTSASSLKALamide (for IP) coupled to keyhole limpet hemocyanin. Anti-HA 12CA5 and anti-Myc 9E10 were purified from mouse ascites as described previously.

##### DNA Constructs

RagA, B, C, and D cDNA clones were purchased from Origene and PCR-cloned into the pEBG and pCMV5-FLAG expression vectors using *Taq* polymerase according to the instructions of the manufacturer (Roche). Mutations were created using a site-directed mutagenesis kit according to the instructions of the manufacturer (Stratagene/Agilent Technologies). To generate pLV-CMV-EGFP-strep-Rag, pEGFP-strept-Rag was constructed first. A Strep tag was first inserted into pEGFP-C1 using annealed oligos at the EcoRI and KpnI sites. RagA and RagC were excised from pCMV5-FLAG-RagA and RagC and subcloned into pEGFP-strept-C1. EGFP-strep-Rag was then PCR-cloned into pLV-CMV. To generate pcDNA1-myc-FLAG-Raptor, a myc tag was inserted into pcDNA1-FLAG-Raptor using annealed oligos with the BamHI and EcoRI restriction sites. The plasmids pCMV5-FLAG-Rheb, pEBG-Rheb WT, pEBG-Ha-Ras G12V, pEBG-Ha-Ras WT, and pEBG-Rap1B are described in Ref. 5. pcDNA1-HA-mTOR, pCMV5-FLAG-mTOR, pMT2-HA-S6Kα1, and pcDNA3-myc-Raptor are described in Ref. 1.

##### Radioisotopes

[α-^32^P]GTP, [γ-^32^P]GTP, inorganic phosphorus-32, and [^3^H]GDP were purchased from PerkinElmer Life Sciences. [^3^H]GDP was purchased from American Radiolabeled Chemicals, Inc.

##### Chemicals and Other Materials

Anti-FLAG® M2 affinity gel (catalog no. A2220) and all chemicals, except as noted, were purchased from Sigma-Aldrich. Acetonitrile (catalog no. A998), hydrochloric acid solution (catalog no. SA56), and Fisherbrand^TM^ pure cellulose chromatography paper (catalog no. 05-714-4) were from Fisher Scientific. Protein G-Sepharose 4 (catalog no. 17-0618) and glutathione-Sepharose 4B (catalog no. 17-0756) were from GE Healthcare Life Sciences. Strep-Tactin Superflow Plus (catalog no. 30004) and tungsten carbide beads (3 mm) (catalog no. 69997) were from Qiagen. TLC PEI cellulose F plates (catalog no. 5725-7) were from EMD Chemicals Inc. The ProteoExtract subcellular proteome extraction kit (catalog no. 539791) was from Calbiochem. DMEM (catalog no. 11995), DPBS^+^Ca^+^ (catalog no. 14040), DMEM phosphate-free (catalog no. 11971), Opti-MEM (catalog no. 31985), sodium pyruvate (catalog no. 11360) (100 mm), minimal essential medium vitamin solution (100×) (catalog no. 11120), minimal essential medium amino acid solution (50×) (catalog no. 11130), minimal essential medium non-essential amino acids (100×) (catalog no. 11140), and l-glutamine (catalog no. 25030) were purchased from Invitrogen.

##### Cell Culture and Transfection

HEK293T, HEK293E, and HeLa cells were cultured in DMEM supplemented with 10% FBS, 100 units/ml penicillin, and 0.1 mg/ml streptomycin in a 5% CO_2_ atmosphere at 37 °C. Transient transfection was performed by lipofection with Lipofectamine or Lipofectamine 2000 according to the instructions of the manufacturer, and the cells were harvested 48 h later with or without stimulation, as indicated in the figures. For amino acid starvation, complete medium was replaced with fresh DMEM (without FBS) for 1 h, and then cells were rinsed twice with DPBS, incubated in DPBS for 1 h, and harvested. To evaluate recovery from AA withdrawal, DPBS-rinsed cells were incubated for 50 min (HEK293T cells) or 1.5 h (HeLa cells) in DPBS. The medium was replaced with fresh DPBS (AA-) or DMEM (AA+) without or with insulin (1 μm, AA+/I) for 10–30 min before harvest.

##### Cell Lysis, Immunoprecipitation, and Strep Tag Pull-down

Cells were frozen with liquid nitrogen, followed by addition of ice-cold lysis buffer. Lysates were subjected to centrifugation at 13,200 rpm for 10 min. Primary antibodies and 30 μl of a 50% slurry of protein G-Sepharose beads were added to the supernatants and incubated with rotation for 2 h at 4 °C. For GST or FLAG and Strep tag pull-down, 30 μl of a 50% slurry of GSH-Sepharose beads, FLAG M2 affinity gel beads, or Strep-Tactin superflow beads, respectively, were added to the supernatants. After 2 h at 4 °C, the immunoprecipitates or the pull-downs were washed sequentially with lysis buffer and wash buffers.

##### Protein Purification

The plasmids pEBG and CMV5-FLAG encoding Rag polypeptides were expressed transiently in HEK293T cells. To retrieve RagA or B/RagC or D heterodimers, GST-tagged RagA or RagB was cotransfected with pCMV5-FLAG-RagC or RagD and harvested 48 h later by extracting frozen cells into ice-cold lysis buffer A (20 mm HEPES (pH 7.4), 2 mm MgCl_2_, 150 mm NaCl, 0.3% CHAPS, 2 mm DTT, and one tablet of EDTA-free protease inhibitor/25 ml of buffer), followed by centrifugation in a microfuge at top speed for 10 min. Supernatants were incubated with GSH-Sepharose beads (2 h at 4 °C) and washed three times with lysis buffer A and three times with wash buffer A (20 mm HEPES (pH 7.4), 2 mm MgCl_2_, and 2 mm DTT). GST-tagged proteins were eluted with GST elution buffer (wash buffer A (pH 8.0) plus 10 mm GSH). For purification of the mTOR/Raptor complex, pcDNA1-myc-FLAG-Raptor was cotransfected with pcDNA1-HA-mTOR or pCMV5-FLAG-mTOR into HEK293T cells and harvested 48 h later into lysis buffer B (50 mm Tris-HCl (pH 7.5), 100 mm NaCl, 10 mm MgCl_2_, 50 mm β-glycerophosphate, 0.3% CHAPS, 1 mm DTT, and one tablet of EDTA-free protease inhibitor/25 ml of lysis buffer). The cell lysates were subjected to anti-FLAG M2 immunoprecipitates and washed three times with lysis buffer B and three times by wash buffer B (50 mm Tris-HCl (pH 7.5), 100 mm NaCl, 10 mm MgCl_2_, 50 mm β-glycerophosphate, and 1 mm DTT), and then the mTOR/Raptor complex was eluted with FLAG elution buffer (wash buffer B containing 100 μg/ml of FLAG peptide).

##### Stable Cell Lines

Lentiviral infection was employed to generate HeLa and HEK293T cell lines stably expressing GFP-tagged Rag variants. Recombinant lentiviruses were generated by Lipofectamine transfection of 4 μg of pLV-CMV, 2 μg of vesicular stomatitis virus spike glycoprotein, and 3 μg of Δ8.9 onto a 10-cm plate of HEK293T cells. The medium was changed to fresh DMEM/10% FBS 24 h later and collected 48 h post-transfection. Debris removed by centrifugation at 230 × *g* for 5 min at room temperature. The viral supernatant was filtered through a 0.45-μm filter and stored at 4 °C. For viral infection, 1000 HeLa cells or HEK293T cells in 0.5 ml of medium, supplemented with Polybrene (6 μg/ml, final) were plated per well of a 24-well plate. After 24 h, the medium was replaced with DMEM (1 ml) containing 1, 10, 100, and 500 μl of viral supernatant and 6 μg/ml Polybrene for a further 24 h. At confluency, recombinant protein expression was estimated as GFP fluorescence using a Nikon Eclipse TE2000-E microscope. Wells with >50% of cells showing GFP expression were expanded progressively and flow-sorted by fluorescence intensity into high, medium, and low GFP expression.

##### [γ-^32^P]GTP/[^3^H]GDP Binding Assays

Binding assays employing [γ-^32^P]GTP or [^3^H]GDP at 25 °C in 40 μl contained purified GST-tagged proteins (0.5∼1 mm, final), 20 mm HEPES (pH7.4), 1∼5 mm EDTA, and 2 mm DTT and were started by addition of guanyl nucleotide to the final concentrations indicated in Fig. 2. Aliquots of 5 μl were removed at various times into 1 ml of ice-cold stop buffer (20 mm Tris-Cl (pH 7.5), 25 mm MgCl_2_, and 100 mm NaCl), and protein-bound nucleotides were recovered after filtration through nitrocellulose membranes and three washes with 4 ml of stop buffer. Radioactivity was determined by liquid scintillation counting. [^3^H]GDP dissociation was measured after equilibration of the Rag proteins with 68 μm (Fig. 2*C*) or 0.5 μm [^3^H]GDP (Fig. 2*D*) for 10 min at 30 °C in 40 μl of binding buffer (as above), followed by addition of 5 μl of a MgCl_2_/nucleotide mixture, yielding a final concentration of (Fig. 2*C*) 20 mm MgCl_2_ and 5 mm ADP or GDP or (*D*) 20 mm MgCl_2_ and 5 μm GTP or GDP. The reactions was stopped at intervals thereafter by addition of 1 ml of stop buffer, followed by nitrocellulose filtration, washing, and determination of protein-bound [^3^H]GDP.

##### Intrinsic GTPase Activity

For estimation of protein-bound [γ-^32^P]GTP, purified GST-tagged proteins (in 40 μl of 20 mm HEPES (pH 7.4), 1 mm EDTA, and 2 mm DTT) were charged with 50 nm of [γ-^32^P]GTP at 25 °C for 10 min. Charging was stopped by adding 9 volumes (360 μl) of GAP assay buffer (20 mm HEPES (pH 7.4), 2 mm MgCl_2_, and 2 mm DTT), and 25 μl of [γ-^32^P]GTP-labeled protein mixture was added to 75 μl of GAP assay buffer at 30 °C containing 0.1 mg/ml of BSA. The reaction was stopped at intervals by adding 1 ml of a stop buffer. Unhydrolysed protein-bound [γ-^32^P]GTP was recovered by nitrocellulose filtration. For the assay by TLC separation of [α-^32^P]GTP, recombinant GTPases were immobilized on 30 μl of a 50% slurry of GSH beads, washed, resuspended in 35 μl of nucleotide charging buffer, and charged with tracer [α-^32^P]GTP so that all nucleotide was bound. After 15 min at 30 °C, 50 μl of an assay buffer (50 mm Tris-HCl (pH 7.5), 100 mm NaCl, 40 mm MgCl_2_, 50 mm β-glycerophosphate, and 1 mm DTT) was added. At the indicated times, ^32^P-labeled nucleotides were extracted and analyzed by TLC as above.

##### GAP Assay

[γ-^32^P]GTP-charged proteins (25 μl) were incubated at 30 °C with 75 μl of GAP assay buffer containing 10 μl of cell lysate for various times (Fig. 3*D*) or with 75 μl of GAP assay buffer containing 5 μl of MonoQ fraction sample for 30 min (*E*). The reaction was stopped by adding 1 ml of stop buffer, and unhydrolysed protein-bound [γ-^32^P]GTP was trapped on nitrocellulose filters. The experiment shown in Fig. 3*D* used a lysate from HEK293T cells prepared with MOPS-based buffers containing EDTA-free protease inhibitor (20 mm MOPS (pH 6.9) and 1 mm DTT) without or with detergent (Detergent-), with 0.3% CHAPS, 1% Triton X-100, or 1% Triton X-100/1% sodium deoxycholate/0.1% SDS. For Fig. 3*E*, an 8 × 10 cm dish of HEK293T cells was lysed into 6 ml of buffer (20 mm Tris-HCl (pH7.6), 1 mm EDTA, 1 mm NaF, 1 mm DTT, and EDTA-free protease inhibitor) with three cycles of freezing and thawing. Supernatants were separated by centrifugation at 13,200 rpm for 10 min by microcentrifuge and filtered by a 0.22-μm filter. Supernatants were separated by a MonoQ 5/50 GL column at a rate of 0.5 ml/min with MonoQ buffer A (20 mm Tris-HCl (pH 7.6), 1 mm EDTA, 1 mm NaF, and 1 mm DTT) and MonoQ buffer B (20 mm Tris-HCl (pH 7.6), 1 mm EDTA, 1 mm NaF, 1 mm DTT, and 1 m NaCl). In Fig. 3*B*, the specificity of the Rag heterodimer was analyzed by incubation with purified maltose-binding protein-tagged Ras GAP NF1. 25 μl of [γ-^32^P]GTP-labeled Rag protein was incubated at 30 °C with 75 μl of GAP assay buffer containing maltose-binding protein-NF1-GRD at a final concentration of 1 nm in 100 μl. The reaction was stopped at intervals by adding 1 ml of charcoal buffer (4% charcoal, 0.1 m HCl, 0.2 mg/ml BSA, 1 mm NaH_2_PO_4_ H_2_O, and 1 mm pyrophosphate), which adsorbs [γ-^32^P]GTP] but not ^32^P_i_. After 15 min at 4 °C, the charcoal mixture was filtered through 0.45 μm membranes, and the ^32^P_i_ was measured by liquid scintillation.

##### Rag-Raptor Binding in Vitro

GST-tagged proteins (approximately 30 pmol, judged by Coomassie Blue stain) immobilized on GSH-Sepharose beads were washed once with lysis buffer B, twice with ice-cold wash buffer C (wash buffer B with 1 mm Mg), once with ice-cold nucleotide charging buffer (50 mm Tris-HCl (pH 7.5), 100 mm NaCl, 5 mm EDTA, 50 mm β-glycerophosphate, 1 mm DTT, and one tablet of EDTA-free protease inhibitor/25 ml of lysis buffer) and resuspended in 0.l ml of nucleotide charging buffer containing 0.2 mm GDP, GTP, or GMP-PNP. After 30 min at 25 °C, 0.9 ml of ice-cold lysis buffer B was added, followed by addition of HA-mTOR/myc-FLAG-Raptor or FLAG-mTOR/myc-FLAG-Raptor (approximately 1 pmol). After 2 h at 4 °C, the beads were washed three times with lysis buffer B, and retained proteins were analyzed by SDS-PAGE and immunoblotting.

##### In Vivo ^32^P Labeling

The determination of Rag-associated ^32^P-guanyl nucleotide in intact cells was performed as described in Ref. 5, with some modifications. HeLa cells (5 × 10^5^) stably expressing GFP-streptag proteins were plated in 6-well culture dishes. After 24 h, the cells were washed and incubated in phosphate-free DMEM containing 0.2 mCi/ml [^32^P]orthophosphate. After 4 h, ^32^P-labeled cells were rinsed and incubated in either homemade P_i_-free DMEM with amino acids (AA+) or the same P_i_-free medium lacking amino acids (AA-), each containing ^32^P_i_ (0.2 mCi/ml). Ninety minutes later, insulin (1.0 μm) was added to some of the cells in DMEM (AA+/I). Thirty minutes later, all cells were rinsed twice with homemade P_i_-free medium (AA- or AA+) lacking ^32^P_i_ and frozen with liquid nitrogen. Cells were lysed into labeling lysis buffer (50 mm HEPES (pH 7.4), 100 mm NaCl, 1 mm KH_2_PO_4_, 1 mm ATP, 5 mm MgCl_2_, 1% TritonX-100, 50 mm β-glycerophosphate, 1 mm NaVO_3_, 10 mg/ml aprotinin, 10 mg/ml leupeptin, and 10 mm benzamidine), the lysates were subjected to centrifugation at 13,200 rpm for 10 min, and Strep-Tactin beads were added to the supernatants. After 1 h at 4 °C, Strep-Tactin-bound proteins were retrieved by centrifugation. The beads were washed three times with labeling wash buffer (50 mm HEPES (pH 7.4), 0.5 m LiCl, 5 mm MgCl_2_, 0.5% Triton X-100, and 0.005% SDS), and the bound nucleotides were eluted into 25 μl of nucleotide elution buffer (5 mm EDTA, 2 mm DTT, 0.2% SDS, 0.5 mm GTP, and 0.5 mm GDP) by incubation at 68 °C for 20 min, followed by centrifugation. The eluates were mixed with methanol (120 μl) and chloroform (60 μl) with vortexing. Phase separation was achieved by the addition of 90 μl of water, vigorous vortexing, and centrifugation for 1 min at 12,000 rpm. The upper phase was dried and resuspended in 15 μl of nucleotide elution buffer.

To estimate the overall HeLa cell GTP/GDP ratio, 0.3 ml of ice-cold acetonitrile, followed by 0.2 ml of ice-cold water, was added to one set of ^32^P-labeled cells expressing GFP-streptag cells and treated as described above for AA-, AA+, and AA+/I. After a second addition and centrifugation of the combined extracts, the supernatants were transferred to a new tube, and the acetonitrile was evaporated. In some experiments, 0.4 ml of ice-cold perchloric acid (0.3 m) was added to each well, and after 10 min on ice, the extracts were centrifuged and the supernatants neutralized with saturated KHCO_3_. Finally, all samples were spotted on glass-backed PEI cellulose TLC plates, dried at room temperature for 1∼2 h, and then the chromatograms were developed in 1.2 m ammonium formate, 0.8 m HCl. The plate was dried for 5 min in an 80 °C incubator, and the ^32^P content of the spots corresponding to GTP and GDP were quantitated by PhosphorImager. The percentage of GTP nucleotide was calculated by [GTP / (GTP + 1.5GDP)].

To examine nucleotide binding of endogenous RagC, HEK293T cells (1 × 10^7^) were grown in 10-cm plates, labeled with [^32^P]orthophosphate, treated as described above for AA- or AA+, rinsed, and frozen with liquid nitrogen. The cells were lysed in a low ionic strength lysis buffer (20 mm HEPES (pH 7.4), 1 mm KH_2_PO_4_, 1 mm ATP, 10 mm MgCl_2_, 1 μm calyculin A, 10 mg/ml aprotinin, 10 mg/ml leupeptin, and 10 mm benzamidine) and cleared by centrifugation. To preclear the supernatants, protein G beads (30 μl of a 50% slurry saturated with nonimmune rabbit IgG) was added and incubated for 1.5 h at 4 °C. Next, 30 μl of a 50% slurry of protein G beads containing prebound nonimmune rabbit IgG or anti RagC antibody (10 μg) was added. After 1.5 h at 4 °C, the beads were washed three times with labeling wash buffer, and bound nucleotides were eluted and analyzed by TLC as above.

To examine the nucleotide binding of Rag heterodimers on the a lysosome-enriched membrane fraction, HEK293T cells (1 × 10^7^) stably expressing GFP-streptag, GFP-streptag-RagA^WT^, GFP-streptag-RagA^T21L^, GFP-streptag-RagC^WT^, and GFP-streptag-RagC^S75L^ were grown on 10-cm plates, labeled with [^32^P]orthophosphate as described above, and harvested at 4 °C without freezing into 1 ml of membrane isolation buffer (20 mm HEPES (pH 7.4), 50 mm KCl, 1 mm EGTA, 5 mm MgCl_2_, 50 mm sucrose, 90 mm potassium gluconate, 5 mm glucose, 2.5 mm ATP, and EDTA-free protease inhibitor). The resuspended cells were mechanically disrupted by TissueLyser (Qiagen) with tungsten carbide 3-mm beads for 6 min at 30/sec frequency. Extracts were centrifuged at 1000 × *g* for 10 min. The supernatant was centrifuged at 16,100 × *g* for 30 min, and the pellet of this centrifugation, which was highly enriched in LAMP2 (see Fig. 7*C*), was mixed with 150 μl of membrane isolation buffer containing 1% Triton X-100. After resuspension, the volume was brought to 1 ml in this buffer and precleared by incubation with protein G beads (30 μl of a 50% slurry) for 2 h at 4 °C. Strep tag proteins were retrieved with Strep-Tactin beads, washed, and then bound ^32^P nucleotides were analyzed after TLC as above.

##### Subcellular Fractionation by Kit (Calbiochem)

One 10-cm plate of HEK293E cells was grown to 90% confluency and then fractionated into cytoplasmic, membrane, nuclear, and cytoskeletal fractions using a subcellular proteome extraction kit according to the instructions of the manufacturer.

##### Immunofluorescence

HeLa cells were plated on noncoated glass coverslips in 6-well tissue culture plates grown to 50% confluency. Cells were deprived of amino acids for 1 h, stimulated with amino acids for 30 min, washed once in PBS, and fixed with 3% paraformaldehyde/2% sucrose in PBS for 10 min. Cells were rinsed twice with PBS and permeabilized with 0.5% Triton X-100 in PBS for 5 min. After rinsing twice with PBS, cells were blocked in 1% BSA/PBS for 10 min and then incubated in 3% BSA/PBS with anti-RagC antibodies for 1 h. Cells were washed three times in PBS and then incubated in 3% BSA/PBS with goat anti rabbit FITC antibodies for 1 h. Cells were then washed three times in PBS and mounted using Vectashield containing DAPI (Vector Laboratories, catalog no. H-1200). Images were captured using a Nikon Eclipse 80i microscope and IPlab software.

##### Sucrose Density Gradient

HEK293T cells (4 × 10 cm plates) were grown to ∼90% confluency, washed, scraped up in ice-cold PBS, pelleted at 230 × *g* for 5 min at 4 °C, and resuspended in 1 ml of homogenization buffer (5 mm Tris-HCl (pH 7.4), 1 mm EDTA, 0.25 m/9% sucrose, 0.25 mm PMSF, and an EDTA-free protease inhibitor mixture tablet (Roche)). Cells were sheared open by four passages through a 25-gauge needle. MgCl_2_ and KCl were then added at a final concentration of 1 mm and 100 mm, respectively. The homogenate was centrifuged at 1000 × *g* for 10 min at 4 °C, and the supernatant was removed on ice. A 10-ml sucrose density gradient of 0.4 m/13%-2.25 m/60% buffered in 10 mm HEPES (pH 7.4) was poured using a gradient maker and 1 ml supernatant layered on the top. The gradient was centrifuged at 100,000 × *g* for 4.5 h at 4 °C in an SW41 rotor in an L8–80 M ultracentrifuge (Beckman). Fractions were then collected in 0.5-ml aliquots from the top of the gradient and subjected to SDS-PAGE and immunoblot analysis.

## RESULTS

### 

#### 

##### Rag Heterodimer Binding and Activation of mTORC1 in Cells

**FIGURE 1.**
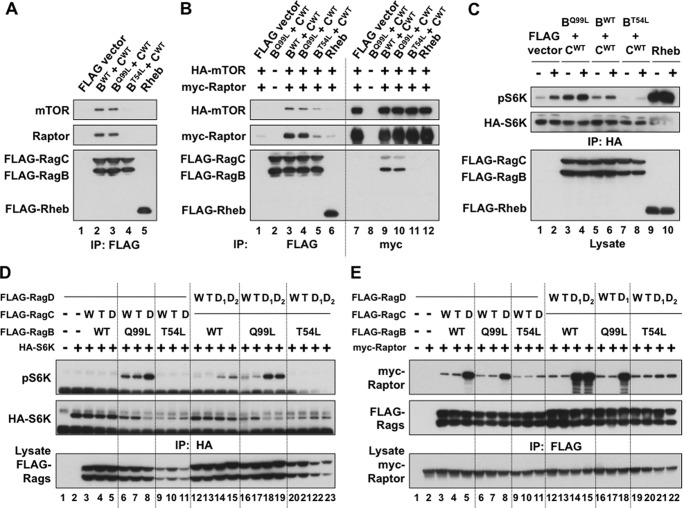
**mTOR complex 1 regulation by and binding to transiently expressed wild-type and mutant Rag heterodimers.** *A* and *B*, binding of endogenous (*A*) and recombinant (*B*) mTORC1 by various RagB/C dimers or Rheb in amino acid-replete cells. The FLAG-tagged RagB and RagC variants indicated or wild-type Rheb were expressed in HEK293T cells together with or without HA-mTOR and myc-Raptor. Anti-FLAG or anti-myc immunoprecipitates (*IP*) were immunoblotted as indicated. *C*, regulation of S6K phosphorylation by coexpressed RagB/C dimers or Rheb with or without amino acid withdrawal. 48 h after cotransfection of HA-S6K with the FLAG-RagB/C dimers indicated or with FLAG-Rheb to HEK293T cells, the medium was replaced by fresh DMEM for 1 h and then by DPBS. After 50 min, the medium of plates AA- was replaced with fresh DPBS, whereas that of plates AA+ was replaced with DMEM, with harvest 10 min thereafter. The extracts and anti-HA immunoprecipitates were immunoblotted for the proteins indicated and for the phosphorylation of HA-S6K. *D*, regulation of S6K phosphorylation by coexpressed RagB/C and RagB/D dimers in amino acid-deprived cells. 48 h after cotransfection of HA-S6K with the FLAG-RagB/C or FLAG-RagB/D dimers indicated, the HEK293T cells were incubated in fresh DMEM for 1 h and deprived of amino acids by DPBS treatment for 1 h. The extracts and the anti-HA immunoprecipitates were immunoblotted for the proteins indicated and for the phosphorylation of HA-S6K. The RagC identified as *D* was S75L, and that identified as *T* was Q120L. The RagD identified as *D_1_* was S76L, and that identified as *D_2_* was S77L, whereas that labeled *T* was Q121L. *E*, the binding of recombinant Raptor to coexpressed RagB/C and RagB/D dimers in amino acid-deprived cells. 48 h after cotransfection of myc-Raptor with the FLAG-RagB/C or FLAG-RagB/D dimers indicated, the HEK293T cells were treated as in *D*, and the extracts and anti-FLAG immunoprecipitates were immunoblotted as indicated. The labeling of RagC and RagD is as in *D*.

Transiently expressed recombinant RagB/RagC heterodimer coprecipitates endogenous (Fig. 1*A*) and recombinant mTOR complex 1 (Fig. 1*B*) specifically and considerably more efficiently than recombinant Rheb. The heterodimers containing wild-type RagB or the RagB switch II mutant (Q99L) retrieve comparable amounts of raptor (Fig. 1, *A* and *B*). However, those containing RagB^Q99L^ cause a substantial phosphorylation of coexpressed S6Kα1 at Thr-389 in amino acid-deprived cells (although less than a comparable expression of Rheb) (Fig. 1*C*). Thus, the greater stimulatory effect of RagB^Q99L^ as compared with RagB^WT^ on mTORC1 signaling is not attributable to increased mTORC1 binding. The stimulatory effect of RagB^Q99L^ on mTORC1 signaling is further augmented if the RagB^Q99L^ is coexpressed with the RagC P-loop mutant (S75L) (Fig. 1*D*, *lane 8 versus lanes 6* and *7*) or the comparable RagD P-loop mutant (S76L or S77L) (*lanes 18* and *19 versus lanes 16* and *17*). RagC^S75L^ has little or no stimulatory effect as compared with wild-type RagC when coexpressed with wild-type RagB in amino acid-deprived cells (Fig. 1*D*, *lane 3 versus lane 5*), whereas both RagD^S76L^ and RagD^S77L^, in complex with wild-type RagB, do stimulate S6Kα1 (Thr-389) phosphorylation (Fig. 1*D*, *lane 12 versus lanes 14* and *15*), suggesting a greater intrinsic potency of RagD as compared with RagC. Regarding the effect of these RagC/D P-loop mutations on the binding of the Rag heterodimer to mTORC1, Rag heterodimers containing RagB^WT^ in complex with the Rag C^S75L^ or RagD^S76L or S77L^ mutants exhibit a greatly increased binding of raptor as compared with Rag heterodimers containing RagB^WT^ in complex with RagC^WT^ (Fig. 1*E*, *lane 3 versus lane 5*) or RagD^WT^ (*lane 12 versus lanes 14* and *15*). A similar pattern is seen with RagB^Q99L^ (Fig. 1*E*, *lane 6 versus lane 8* and *lane 16 versus lane 18*). Thus, it is likely that the stimulatory effects of the RagC^S75L^/RagD^S76L^/RagD^S77L^ mutations on S6K1 phosphorylation are attributable, in large part, to enhanced Rag binding to raptor. Rag heterodimers containing the RagB P-loop mutant (T54L) together with any RagC (or RagD, not shown) variant act as dominant inhibitors of S6Kα1 (T389) phosphorylation in amino acid-replete cells (Fig. 1*C*). Notably, the RagB^T54L^ mutant suppresses the enhanced binding of raptor to Rag heterodimers containing RagC^S75L^ (Fig. 1*E*, *lanes 5* and 8 *versus lane 11*) or RagD^S76L^ (*lanes 14*, *15*, and *18 versus* lanes *21* and *22*).

On the basis of the inhibitory effects of the RagA P-loop mutant (T21L, the residue homologous to RagB^T54^) and RagA switch I mutations on raptor binding and S6K1 activation, it has been proposed previously that the primary interaction surface of the Rag heterodimer with raptor is through the RagA-B subunit (19, 20). These results are compatible with this conclusion. Nevertheless, the stimulatory effect of RagC/D P-loop mutation on Rag heterodimer binding to raptor is impressive and, together with the inability of single RagA/B/C/D polypeptides to regulate raptor (not shown), emphasizes the importance of both heterodimer partners in configuring the raptor binding site.

Current concepts concerning the role of Rag guanyl nucleotide charging on the regulation of raptor binding and the way that Rag P-loop and switch II mutations alter Rag function rely heavily on extrapolation from the behavior of Ras GTPase. Effector binding to Ras is determined by the extent of Ras GTP charging (21). The Ras^S17N^ P-loop mutation results in GDP-only binding and loss of effector binding, leading to the inference that the homologous Rag P-loop mutants also bind GDP preferentially and that the diminished binding of raptor to RagA^T21L^/RagB^T54L^ is presumed because of the replacement of GTP by GDP. The paradoxically enhanced binding of raptor to the comparable RagC/D P-loop mutants (also presumably GDP-charged) is explicable as an indirect action of the RagC/D mutant on the configuration of the RagA/B partner. Extending this reasoning, the ability of amino acid sufficiency to stimulate mTORC1/raptor binding to the wild-type Rag heterodimer in cells has been plausibly attributed to an ability of amino acids to regulate the guanyl nucleotide charging of the Rag heterodimers, either by promoting GTP binding to the RagA/B partner or, alternatively, enhancing the GDP content of the RagC/D partner or both. We sought to examine these inferences by characterizing the guanyl nucleotide binding of wild-type and mutant Rags *in vitro* and in cells and the effect of guanyl nucleotides on Rag binding to raptor *in vitro*.

##### Rag Heterodimer Guanyl Nucleotide Binding in Vitro

**FIGURE 2.**
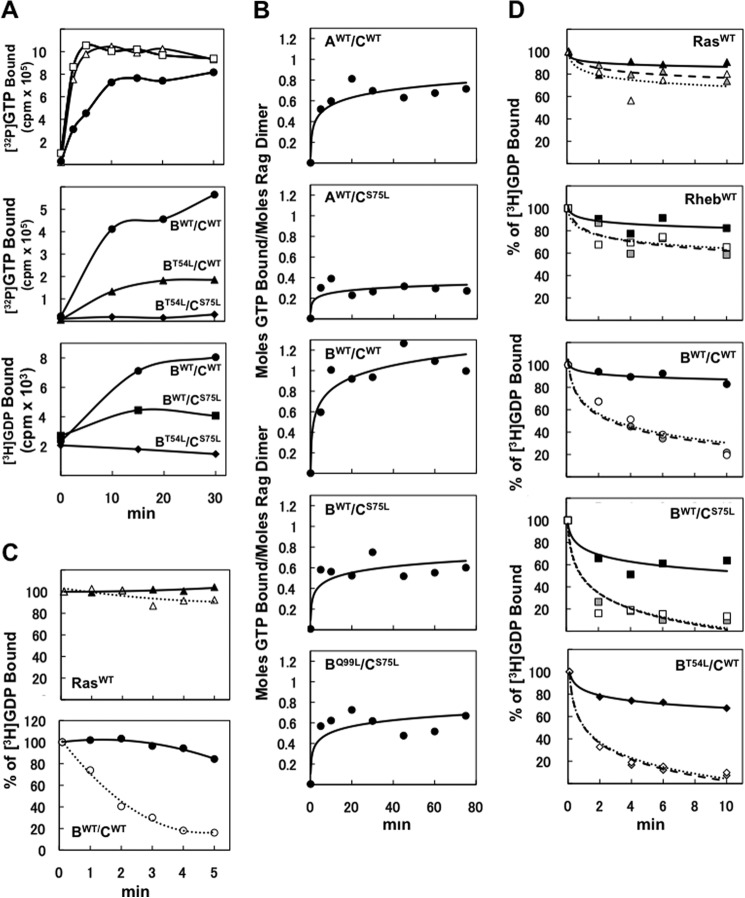
**Guanyl nucleotide binding to and dissociation from Rag heterodimers.** *A*, time course of guanyl nucleotide tracer binding to variant RagB/C heterodimers. Equal Coomassie Blue-stained amounts of GST-Ha-Ras (▵, *top panel*), GST-Rheb (□, *top panel*), GST-RagB/FLAG-RagC heterodimers (B^WT^/C^WT^, ●; B^T54L^/C^WT^, ▴; B^T54L^/C^S75L^, ♦; B^WT^/C^S75^, ■) were incubated with [γ^32^P]GTP (50 nm, *top* and *center panels*) or [^3^H]GDP (68 μm, *bottom panel*) at 30 °C. *B*, stoichiometry of guanyl nucleotide binding to variant RagA or B/C heterodimers. Various GST-RagA or B/FLAG-RagC heterodimers were incubated with 0.1 mm [γ^32^P]GTP at 25 °C. The ordinate indicates the moles GTP bound per dimer. *C*, wild-type RagB/C heterodimers exhibit rapid spontaneous exchange of bound [^3^H]GDP. Equal Coomassie Blue-stained amounts of GST-Ha-Ras (*top panel*) and wild-type GST-RagB/FLAG-RagC heterodimer (*bottom panel*) were preincubated with [^3^H]GDP (68 μm) to a steady state at 30 °C. At t = 0, nonradioactive ADP (5 mm, ▴ and ●) or GDP (5 mm, ▵ and ○) was added, and the protein-bound [^3^H]GDP was measured at intervals thereafter. *D*, [^3^H]GDP bound to wild-type RagB or RagC within heterodimers is displaced by GTP and GDP with similar efficacy. GST-Ha-Ras (*first panel*), GST-Rheb (*second panel*), and various GST-RagB/FLAG-RagC heterodimers were incubated with 0.5 μm [^3^H]GDP to a steady state at 25 °C. At t = 0, water (▴, ■, ●, and ♦), nonradioactive GTP (5 μm, *gray symbols*), or GDP (5 μm, ▵, □, ○, and ♢) were added, and the protein-bound [^3^H]GDP was measured at intervals thereafter.

We examined the ability of the purified epitope-tagged wild-type and mutant Rag heterodimers, transiently expressed and purified from HEK293T cells, to bind, release, and hydrolyze guanyl nucleotides in comparison with Ras or Rheb. The recombinant wild-type RagB/C heterodimer binds [γ-^32^P]GTP rapidly in a manner similar to recombinant c-Ha-Ras or Rheb (Fig. 2*A*, *top* and *center panels*) and binds [^3^H]GDP (*bottom panel*) as well. Regarding the ability of the mutant Rags used for functional studies to bind guanyl nucleotides, we first examined the Rags mutated at the site homologous with Ras^Ser-17^, *i.e.* RagB^T54L^ and RagC^S75L^ (Fig. 2*A*, *top* and *bottom panels*, and *B*). When either mutant was paired with a wild-type heterodimer partner, the heterodimer containing the mutant partner bound roughly half as much tracer [γ-^32^P]GTP (Fig. 2*A*, *center panel*) or [3H]GDP (*bottom panel*) as a wild-type RagB/C heterodimer. The RagB^T54L^/RagC^S75L^ heterodimer was entirely unable to bind [γ-^32^P]GTP or [^3^H]GDP. Moreover, at 100 μm GTP (corresponding approximately to the intracellular GTP concentration), the binding of GTP to wild-type RagA or B/C heterodimers plateaus at a stoichiometry of ∼0.6–1.0 mol/mol Rag dimer (Fig. 2*B*, *first* and *third panels*). The wild-type heterodimers bind approximately twice as much GTP as Rag heterodimers containing a wild-type RagA or B with a RagC P-loop mutant (S75L), confirming the inability of RagC^S75L^ to bind guanyl nucleotide and indicating that the P-loop mutation in the RagC heterodimer partner does not affect nucleotide binding to a wild-type RagA/B partner at saturating levels of GTP (Fig. 2*B*). Thus, in contrast to Ras^S17N^, which exhibits a nearly 1000-fold decrease in affinity for GTP with only a mildly reduced affinity for GDP (16), the corresponding Rag P-loop mutants appear unable to bind either GDP or GTP. The RagB^Q99L^ mutant corresponding to Ras^Q61L^, when partnered with a RagC P-loop mutant (S75L), binds [γ-^32^P]GTP to an extent indistinguishable from a RagB^WT^/C^S75L^ heterodimer (Fig. 2*B*, *fifth panel*).

We next examined the rate of dissociation of guanyl nucleotide, comparing the Rag heterodimers with Ras (Fig. 2*C*). Wild-type Ha-Ras-bound [^3^H]GDP exhibits little or no dissociation upon addition of ADP or GDP (+20 mm Mg). In contrast, GDP, but not ADP, causes a rapid dissociation of wild-type RagB/C heterodimer-bound [^3^H]GDP with *t*_½_ of 1–2 min at 30 °C (Fig. 2,*C*, *bottom panel*, and *D*). [^3^H]GDP bound to RagB^WT^/RagC^WT^, RagB^WT^/RagC^S75L^, or RagB^T54L^/RagC^WT^ is displaced comparably by excess GDP or GTP (Fig. 2*D*). The rapid spontaneous dissociation of guanyl nucleotide from Rag heterodimers indicates that guanyl nucleotide exchange is unlikely to be a rate-limiting step for the conversion of RagB to the GTP-bound state.

**FIGURE 3.**
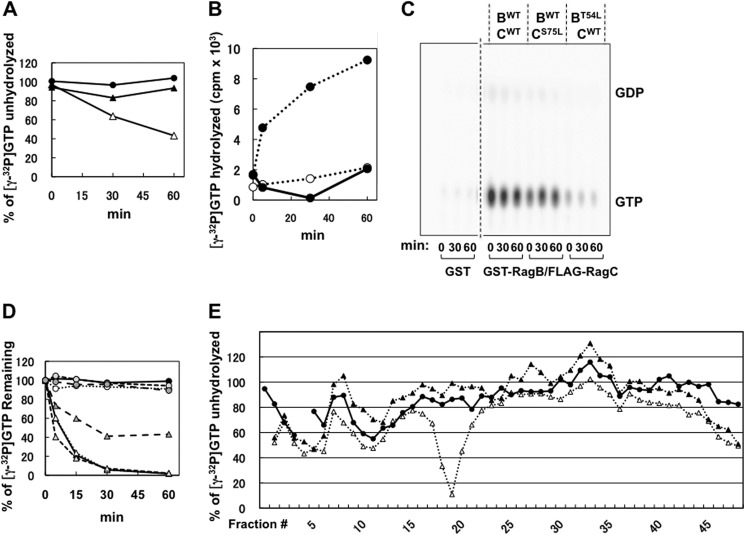
**Rag heterodimers lack detectable GTPase activity *in vitro*.** *A*, the RagB/C wild-type heterodimer does not hydrolyze [γ-^32^P]GTP *in vitro*. GST-Ha-Ras^WT^ (▵), GST-Ha-Ras^G12V^ (▴), and GST-RagB^WT^/FLAG-RagC^WT^ (●) were charged with [γ-^32^P]GTP and examined for intrinsic GTPase activity. The amount of unhydrolysed [γ-^32^P]GTP on proteins was measured at intervals thereafter by nitrocellulose filter binding assay. *B*, a recombinant catalytic fragment of NF1 stimulates GTP hydrolysis by Ha-Ras^WT^ but not Ha-Ras^G12V^ or RagB^WT^/C^WT^. GST-Ha-Ras^WT^ (●, *dashed line*), GST-Ha-Ras^G12V^ (○, *dashed line*), or GST-RagB^WT^/FLAG-RagC^WT^ (●, *solid line*) were charged with [γ-^32^P]GTP, and a catalytic fragment of NF1 was added. The release of ^32^P_i_ at 30 °C was measured at intervals thereafter by the charcoal method. *C*, neither RagB wild-type or RagC wild-type within a heterodimer hydrolyze [α-^32^P]GTP detectably *in vitro*. GST and various GST-RagB/FLAG-RagC heterodimers were charged with [α-^32^P]GTP, and at intervals thereafter, nucleotides were extracted and separated on TLC on PEI cellulose. *D*, extracts from HEK293T cells do not promote the ability of RagB/C to hydrolyze [γ-^32^P]GTP. HEK293T cells were lysed in the absence of detergent (*solid line*) or with CHAPS (0.3%, *dotted line*), TritonX-100 (1%, *short dashes*), or RIPA buffer (Pierce) (*long dashes*). GST-Ha-Ras^WT^ (*triangles*) and GST-RagB^WT^/FLAG-RagC^WT^ (*circles*) charged with [γ-^32^P]GTP were mixed with each of these extracts at 30 °C, and the amount of protein-bound [γ-^32^P]GTP was measured by filtration through nitrocellulose filters. *E*, MonoQ anion exchange chromatography of a HEK293T cell extract contains GAP activity toward Ha-Ras wild-type, not RagB/C wild-type. HEK293T cells were extracted by freezing and thawing and separated by anion exchange chromatography. Each fraction was incubated at 30 °C with GST-Ha-Ras^WT^ (▵, *dashed lines*), GST-Ha-Ras^G12V^ (▴, *dashed lines*), and GST-RagB^WT^/FLAG-RagC^WT^, each charged with [γ-^32^P]GTP. After 30 min, protein-bound [γ-^32^P]GTP was measured as in *C*.

Although wild-type Ha-Ras has a low intrinsic GTPase activity, this is completely abolished in the Ras^G12V^ mutant (Fig. 3*A*). Moreover, the increased GTPase activity caused by addition of the catalytic domain of Ras GAP NF1 to wild-type Ha-Ras is entirely absent in the Ras^G12V^ mutant (Fig. 3*B*). Likewise, conversion of [^32^P]GTP to [^32^P]GDP by the wild-type RagB/C heterodimer is not detectable in the absence (Fig. 3, *A–C*) or presence (*B*) of NF1. Therefore, we sought evidence for the presence of a Rag-GTPase-activating activity in cell extracts. Incubation of [γ-^32^P]GTP-loaded RagB/C with extracts prepared from amino acid-deprived HEK293T cells (Fig. 3*D*) with homogenates of mouse liver or brain and various membrane fractions from these sources (not shown), treated with or without nonionic or ionic detergents, or after brief treatment *in vitro* with trypsin (1–10 μg/ml), did not cause detectable hydrolysis of RagB/C-bound [γ-^32^P]GTP, whereas most of these extracts caused a readily measurable increase in the rate of hydrolysis of Ras-bound GTP (*D*). Similarly, chromatographic separation of cytosolic extracts from amino acid-deprived HEK293T cells by anion exchange gave a clear major peak of Ras-GAP activity, whereas no such peak was evident with a RagB/C-GTP substrate (Fig. 3*E*), nor was Rag-GAP activity evident in cation exchange chromatography (not shown). Thus, we are unable to detect an intrinsic RagB/C GTPase during incubations of the purified Rags up to 1 h at 30° *in vitro* or a RagB/C GTPase-activating protein.

##### Rag Heterodimer Binding to mTORC1 in Vitro Is Guanyl Nucleotide-independent

**FIGURE 4.**
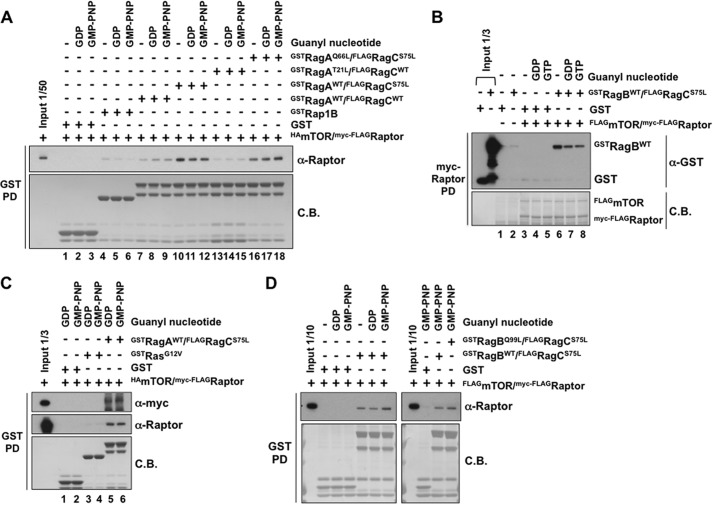
**The binding of mTOR complex1 to various RagA or B/C heterodimers *in vitro*.** *A*, variant RagA/C heterodimers bind mTOR complex1 specifically and in a guanyl nucleotide-independent manner. Equal amounts of GST tagged proteins indicated were immobilized on GSH-Sepharose; loaded with 0.2 mm GDP, GMP-PNP, or water; and incubated with purified HA-mTOR/myc-FLAG-Raptor. The bound Raptor was analyzed by immunoblotting, and GST-proteins were detected by Coomassie Blue staining (*C.B.*). *PD*, pull-down. *B*, immobilized mTOR complex1 binds GST-RagBWT/FLAG-RagC^S75L^ in a guanyl nucleotide-independent manner. Coexpressed FLAG-mTOR/myc-FLAG-Raptor was isolated using Sepharose-bound anti-myc and mixed with GST or GST-RagB^WT^/FLAG-RagC^S75L^ charged with 0.2 mm GDP, GTP, or water. After washing, the Sepharose-bound proteins were analyzed by anti-GST immunoblot analysis and Coomassie Blue staining. *C*, GST-RagA/RagC^S75L^, but not GST-HA-Ras^G12V^, binds mTOR complex 1 *in vitro*. GST, GST-RagA/RagC^S75L^, and GST-HA- Ras^G12V^ immobilized on GSH-Sepharose were charged with 0.2 mm GDP or GMP-PNP and incubated with recombinant HA-mTOR/myc-FLAG-Raptor. After incubation and washing, the retained proteins were analyzed by immunoblot analysis for myc (*top panel*) and Raptor (*center panel*) and by Coomassie Blue staining. *D*, the constitutively active GST-RagB^Q99L^/FLAG-RagC^S75L^ heterodimer and the minimally active GST-RagB^WT^/FLAG-RagC^S75L^ heterodimer bind mTOR complex1 to a similar extent *in vitro*. GST or the GST-Rag heterodimers immobilized on GSH-Sepharose were charged with 0.2 mm GDP, GMP-PNP, or water and incubated with recombinant FLAG-mTOR/myc-FLAG-Raptor. After incubation and washing, the retained proteins were immunoblotted with anti-myc and stained with Coomassie Blue.

Recombinant Rag heterodimers, expressed with amino terminal FLAG or GST fusions, were purified by the epitope after transient expression in HEK293T cells and charged *in vitro* with nonradioactive GDP or GMP-PNP (0.2 mm). Epitope-tagged raptor, coexpressed with mTOR, was affinity-purified, eluted, and mixed with an excess of immobilized GST or the GST-Rag heterodimer. As seen in Fig. 4*A*, Raptor binds to the GST-Rag A/C heterodimer but not to GST. The RagA/C wild-type heterodimer binds raptor to a similar extent, whether nucleotide-free or charged with either GDP or GMP-PNP. Specific, nucleotide-independent binding of GST-Rag heterodimers, but not of GST to raptor, is also evident if the raptor, rather than GST-Rag, is immobilized (Fig. 4*B*). Rap1B^WT^ (Fig. 4*A*) or Ha-Ras^G12V^ (Fig. 4*C*), immobilized at levels comparable with the Rag heterodimer partners, bind little or no raptor as compared with Rag heterodimers, providing additional evidence of the specificity of raptor binding to the Rag heterodimers *in vitro*. Rag heterodimers containing wild-type RagA/B and RagC^S75L^ also bind raptor in a nucleotide-independent manner but to an extent greater than wild-type Rag heterodimers (Fig. 4*A*), mirroring the effect of the RagC^S75L^ mutation of raptor binding in cells (Fig. 1*D*). The RagA^Q66L^ mutation does not alter the extent of raptor binding (Fig. 4*A*, compare *lanes 10–12* to *lanes 16–18*), as also seen when comparing RagB WT with Q99L (Fig. 4*D*). In contrast, RagA^T21L^ partnered with RagC^WT^ binds less raptor as compared with RagA/C wild-type (Fig. 4*A*, *lanes 7–9 versus lanes 13–15*), mimicking the behavior in cells of this RagA P-loop mutant (Fig. 1, *A* and *B*). In summary, Rag heterodimers bind raptor *in vitro* to a similar extent, whether the RagA/B partner is nucleotide-free or GDP- or GTP-charged. Conversely, mutation of RagC P-loop (S75L) enhances, whereas mutation of RagA P-loop (T21L) depresses, raptor binding *in vitro* to the corresponding Rag heterodimers, a behavior that parallels the effect of these mutations on raptor binding during transient expression in cells. The significant effects of Rag P-loop mutation on raptor binding in cells and *in vitro*, together with the insensitivity of raptor binding *in vitro* to changes in RagA/B guanyl nucleotide content, suggests that the effects of Rag P-loop and switch II mutations on Rag regulation of mTORC1 are independent of any effects on Rag nucleotide binding.

##### Rag Heterodimer Binding of Guanyl Nucleotide in Cells

**FIGURE 5.**
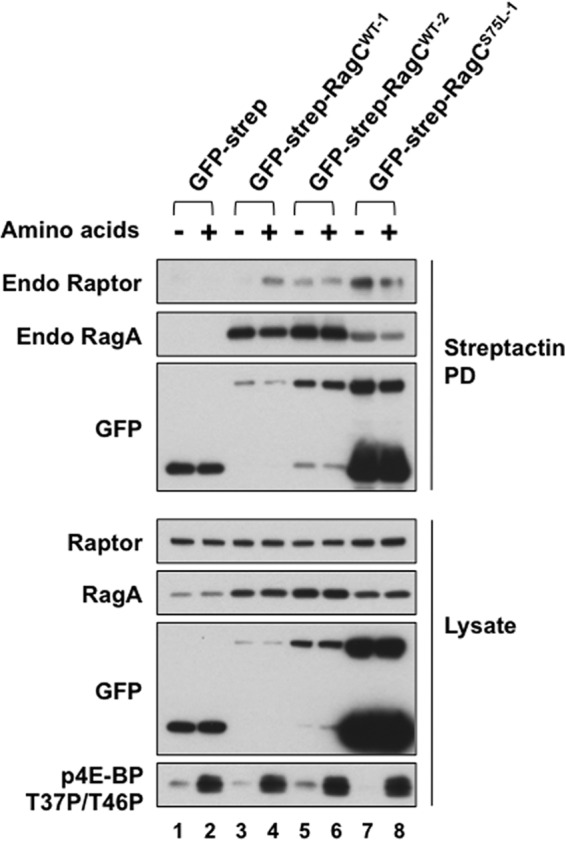
**The amino acid dependence of mTOR complex 1 binding to recombinant Rag heterodimers in HeLa cells stably expressing GFP-streptag-RagC variants at differing abundances.** HeLa cells stably expressing GFP-streptag, GFP-streptag-RagC^WT^, or GFP-streptag-RagC^S75L^ were selected by cell sorting for GFP abundance, and the expression of the full-length recombinant protein was estimated by GFP immunoblot analysis of lysates. The cells were incubated in fresh DMEM for 1 h and then deprived of amino acids by incubation in DPBS for 1.5 h. The medium of plates AA- was replaced with fresh DPBS, whereas that of plates AA+ was replaced with DMEM, with harvest 10 min thereafter. Strep-Tactin pull-downs (*PD*) and the lysates were analyzed by immunoblotting for endogenous (*Endo*) Raptor and RagA, for GFP, and for 4E-BP(T37P/T46P).

We next examined the state of Rag GTP charging in intact cells. HeLa and HEK293T cell lines that stably express GFP-streptag fused to the amino termini of RagC or RagA wild-type polypeptides and to the RagC^S75L^ or RagA^T21L^ mutant polypeptides. Control lines expressed GFP-streptag alone. Inasmuch as RagA^T21L^ and RagC^S75L^ do not bind nucleotide, the guanyl nucleotide retrieved with the P-loop mutant Rags can be attributed to the endogenous Rag heterodimer partner. Fig. 5 demonstrates some effects of recombinant RagC wild-type and S75L stably overexpressed at several levels of abundance. Immunoblot analysis of HeLa lysates with an antibody directed to an epitope shared by RagA and RagB demonstrates that RagA is far more abundant (not shown). Note that stable overexpression of recombinant GFP-strep-RagC is accompanied by an increased abundance of endogenous RagA (Fig. 5, *Lysate*, *second panel*) but does not alter the abundance of raptor. Strep-Tactin pull-downs of GFP-streptag-RagC (Fig. 5, *Streptactin PD panels*) retrieve endogenous RagA as expected, although recovery of RagA with GFP-streptag-RagC^S75L^ is less efficient than with RagC^WT^. Amino acid withdrawal does not alter the amount of RagA retrieved with GFP-strep-RagC. In the HeLa cell line designated strept-GFP-RagC^WT-1^, endogenous raptor is retrieved by Strep-Tactin in an amino acid-dependent manner, confirming earlier results (12). However, the same strept-GFP-RagC^WT^ polypeptide expressed at a higher level (designated strept-GFP-RagC^WT-2^) binds endogenous raptor in a constitutive, amino acid-independent manner. Strept-GFP-RagC^S75L^ expressed at an even higher level also exhibits constitutive, amino acid-independent binding of endogenous raptor. Although overall pull-down of raptor is diminished comparing GFP-streptag-RagC^S75L^ with strept-GFP-RagC^WT^, the efficiency of raptor retrieval, as judged by the concomitant retrieval of endogenous RagA, is higher for GFP-streptag-RagC^S75L^ (Fig. 5, *Streptacin PD*, *first* and *second panels*). Importantly, overexpression of GFP-streptag^WT^ or GFP-streptag-RagC^S75L^ does not significantly alter the inhibition or restoration of 4E-BP[Thr-37/Thr-46] phosphorylation engendered by amino acid withdrawal and readdition (Fig. 5, *bottom panel*). Inasmuch as amino acid sufficiency does not alter Rheb GTP charging (9, 11), this indicates that there are one or more additional, necessary regulatory steps, other than raptor binding to Rag, between amino acids and mTORC1 activation that are altered minimally, if at all, by Rag overexpression at these levels. This is consistent with the subsidiary role envisaged for the Rags, whose binding of raptor is necessary, but not sufficient, for mTORC1 activation unless mTORC1 is effectively approximated with Rheb-GTP.

**FIGURE 6.**
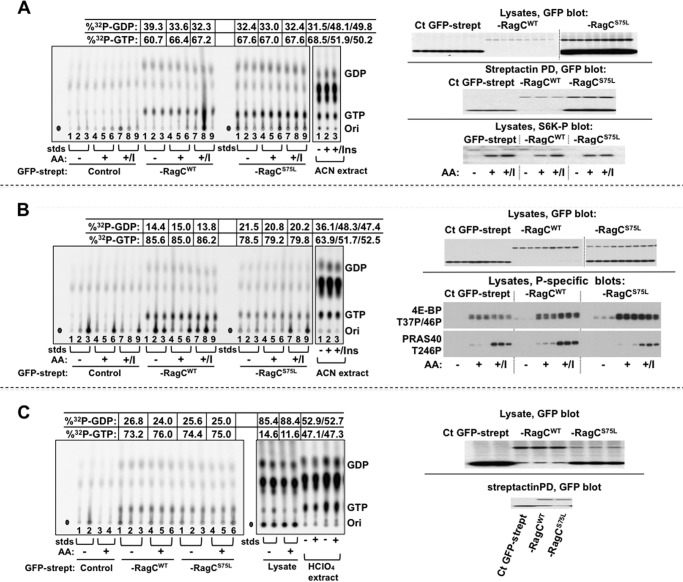
**The effect of amino acid withdrawal and insulin on the [^32^P]guanyl nucleotide content of stably expressed RagC^WT^ and RagC^S75L^ heterodimeric complexes in ^32^P_i_-labeled HeLa cells.** *A*, *B*, and *C*, replicate plates of HeLa cells stably expressing GFP-streptag, GFP-streptag-RagC^WT^, or GFP-streptag-RagC^S75L^ were incubated in P_i_-free DMEM containing ^32^P_i_ (0.2 mCi/ml). After 4 h, the cells were rinsed and incubated in either homemade P_i_-free medium (*AA*+) or P_i_-free medium lacking amino acids (*AA*-), each containing ^32^P_i_ (0.2 mCi/ml), for another 2 h. In *A* and *B*, insulin (1.0 μm) was added to some of the cells in DMEM (*AA*+/*I*) 30 min before harvest. The nucleotides bound to Strep-Tactin (*strept*) pull-downs were extracted and separated by TLC on PEI cellulose. One set of ^32^P -labeled HeLa cells expressing FP-streptag, treated as described above for AA-, AA+, and AA+/I, were rinsed, extracted directly into acetonitrile, and then the solubilized total nucleotides were separated by TLC. The ^32^P comigrating with GDP and GTP was quantitated by phosphorimaging. After subtraction of the averaged values found in the GFP-streptag lanes, the percentage of [^32^P]GTP was calculated as [[^32^P]GTP / (1.5 × [^32^P]GDP + [^32^P]GTP)], and the averaged values are shown. In *A*, the *top* and *center panels* on the *right* show GFP immunoblot analyses of the cell lysates and representative Strep-Tactin pull-downs (corresponding to *lanes 1*, *4*, and *7* of each set). The *bottom panel* shows an immunoblot analysis of S6K(T389P) corresponding to *lanes 1*, *4*, and *7* of each set. In *B*, the *top panel* on the *right* shows a GFP immunoblot analysis of the lysates, whereas the *center* and *bottom panels* show lysate immunoblot analyses for 4E-BP(T37P/T46P) and PRAS40(S246P), respectively. In *C*, one set of ^32^P-labeled HeLa cells expressing GFP-streptag, treated as in Fig. 6, were rinsed, extracted directly into HClO_4_ (0.3 m, 0 °C, *HClO_4_ extract*). The HClO_4_ supernatants were neutralized with KHCO_3_, and the [^32^P]guanyl nucleotides were quantified as above. In addition, the nucleotides in the lysate after pull-down of the Strep-Tactin beads were also analyzed. Immunoblot analyses of the lysates and representative Strep-Tactin pull-downs are shown in the *right panel. Ori*, origin; *std*, guanyl nucleotide standards; *Ins*, insulin; *ACN*, acetonitrile; *Ct*, C-terminal.

To determine the effects of amino acid sufficiency on Rag guanyl nucleotide charging, cells were labeled with ^32^P_i_ (0.2 mCi/ml) for 4 h in the presence of P_i_-free DMEM containing 10% dialyzed calf serum. Thereafter, the cells were washed in the serum-free medium used for the next phase of incubation (either P_i_ and amino acid-free medium or P_i_-free, amino acid-containing medium) followed by readdition of either P_i_ and amino acid-free medium with ^32^P_i_ or P_i_-free, amino acid-containing medium with ^32^P_i_ (^32^P_i_ conditions as in the first incubation). After 2 h, the medium was removed, and the cells were rapidly rinsed at 4 °C and extracted into a buffer containing magnesium. Strep-Tactin-bound proteins were isolated and washed. The nucleotides were extracted, mixed with nonradioactive GTP and GDP, and separated by TLC on PEI cellulose. The nonradioactive GTP/GDP spots were identified, and comigrating ^32^P was quantified by PhosphorImager. Aliquots of the original extracts and the supernatant of the Strep-Tactin pull-downs were extracted directly into acetonitrile to retrieve total ^32^P nucleotides. As shown in Fig. 6, ∼60–85% of [^32^P]GDP+GTP bound to both the RagA endogenous/GFP-streptag-RagC^WT^ and to the RagA endogenous/GFP-streptag-RagC^S75L^ is [^32^P]GTP, and the fractional content of [^32^P]GTP is not altered by removal of amino acids for 2 h prior to extraction.

The regulation of mTORC1 signaling in these experiments was examined. The regulation of S6K(Thr-389) phosphorylation is not detectably different in the three GFP-streptag-expressing cell lines. Transfer of cells into amino acid-free medium for 2 h before harvest abolishes S6K(Thr-389) phosphorylation as compared with cells incubated in amino acid-containing medium, and phosphorylation is slightly increased by the further addition of insulin (Fig. 6*A*). The phosphorylation of 4E-BP(Thr-37/46) is altered in a subtle manner. After 2 h, amino acid withdrawal 4E-BP(Thr-37/46) phosphorylation is very low or absent in the control (GFP-streptag) cells, whereas it is detectably greater in the cells expressing GFP-streptag-RagC^WT^ and increased further in the cells expressing GFP-streptag-RagC^S75L^ (Fig. 6*B*). Readdition of amino acids gives robust phosphorylation of 4E-BP(Thr-37/46) in all three cell lines. PRAS40(Thr-246) phosphorylation, which is catalyzed by Akt, is responsive to insulin rather than amino acids.

Regarding the whole cell GTP/GDP+GTP ratio, deproteinated extracts prepared by direct addition of acetonitrile to ^32^P-labeled cells were examined by TLC on PEI cellulose. The ^32^P-labeled spots comigrating with GTP and GDP appear to be clearly separated, and the ratio of [^32^P]GTP/(1.5 × [^32^P]GDP + [^32^P]GTP) in the extract from amino acid-deprived cells is estimated to be ∼60–70%. This ratio is well within the range of those reported for a variety of cell and tissue sources (22). Nevertheless, the calculated extract ratio, which is very similar to that of the GFP-streptag-RagC-bound guanyl nucleotides, may be low because of contamination of the GDP spot by ^32^P-labeled cellular components other than [^32^P]GDP. Note that amino acid readdition increases the ^32^P comigrating [^32^P]GDP without alteration in the [^32^P]GTP spots, resulting in an apparent decrease in the calculated fractional content of [^32^P]GTP. We are uncertain whether this reflects an amino acid-induced increase in [^32^P]GDP content or some comigrating ^32^P-labeled metabolite. We have been unable improve our estimate of extract fractional GDP/GTP content using chromatographic methods because sufficient separation of GDP from more abundant *A*_260_ absorbing substances were not achieved. Nevertheless, it is notable that, during the incubation of the cell extracts for pull-down of the GFP-streptag complexes, extensive hydrolysis of lysate [^32^P]GTP occurs so that the fractional content of lysate [^32^P]GTP at the time of Strep-Tactin harvest is reduced to 10–15% (Fig. 6*C*). Thus, the finding that the guanyl nucleotide recovered with the RagC complexes is ∼60–85% [^32^P]GTP strongly reinforces the conclusion that the RagA/B-bound GTP does not undergo significant exchange with lysate guanyl nucleotides or hydrolysis *in vitro* and is actually protected from GTPase activity, both in the cell and in the extract, as inferred from the *in vitro* experiments described in Fig. 3. Thus, the fractional GTP charging of endogenous RagA in complex with GFP-streptag-RagC is constitutively very high, probably equivalent to the ratio of cellular GTP/GDP+GDP, and is not altered by amino acid sufficiency or insulin.

**FIGURE 7.**
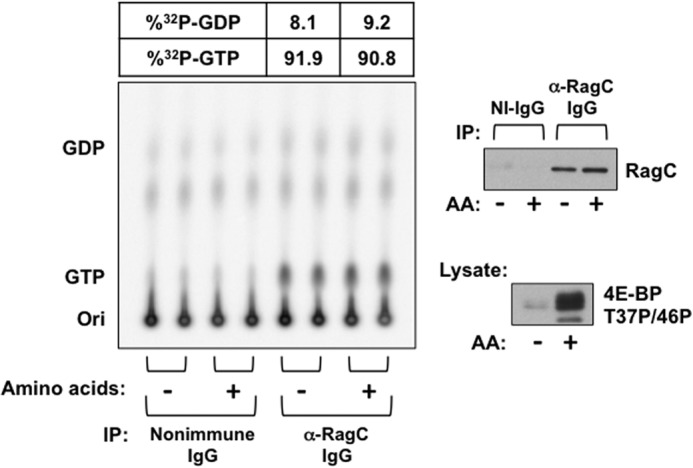
**Effect of amino acid withdrawal on [^32^P]guanyl nucleotide content of RagC-containing heterodimers endogenous to ^32^P_i_-labeled HEK293T cells.** Replicate plates of HEK293T cells were incubated in P_i_-free DMEM containing ^32^P_i_ (0.2 mCi/ml). After 4 h, the cells were rinsed and incubated in either homemade P_i_-free medium (*AA*+) or P_i_-free medium lacking amino acids (*AA*-), each containing ^32^P_i_ (0.2 mCi/ml). Cells were extracted 2 h later, and immunoprecipitation (*IP*) was performed using nonimmune (*NI*) rabbit IgG or anti-RagC IgG. Nucleotides were extracted from the washed immunoprecipitates and separated by TLC on PEI cellulose. An immunoblot analysis of the extract for 4E-BP(T37P/T46P) and of the immunoprecipitates for RagC are shown in the *right panels. Ori*, origin.

We sought to confirm that the GTP charging of the recombinant RagC heterodimers reflects that of the endogenous complexes. However, the amounts of endogenous RagC retrieved from HeLa cells using anti-RagC antibodies were inadequate. A sufficient yield of endogenous RagC was recovered from ^32^P-labeled HEK293T cells. In a single experiment, we found the fractional content of [^32^P]GTP bound to the endogenous complexes to be ∼90% and unaffected by amino acid withdrawal (Fig. 7). This estimate may be somewhat high because of the very small difference in ^32^P comigrating with GDP in the anti-RagC and control immunoprecipitates. Nevertheless, withdrawal of amino acids is clearly without effect.

##### Nucleotide Charging of Membrane-bound Rag Heterodimers

Sabatini and coworkers (17, 18) reported that the ability of amino acids to activate mTORC1 through the Rag heterodimers is critically dependent on the binding of the latter to a complex of five proteins they called the “Ragulator” that is localized to the cytoplasmic face of the lysosome. The protein p18/LAMTOR1 is attached to the cytoplasmic surface of the lysosome through amino-terminal myristoylation/palmitoylation (23, 24) and serves as a scaffold for the rest of the Ragulator complex. Loss or depletion of p18/LAMTOR1 eliminates raptor and Rag localization to the lysosome and prevents amino acid activation of mTORC1, emphasizing the importance of late endosomal/lysosomal localization for the amino acid regulation of mTORC1. Appending the Rheb C-terminal 15 amino acids (containing the C*AAX* and other localization signals) to raptor eliminates the requirement for the Rags and the Ragulator (17). mTORC1 is thereby activated and rendered insensitive to amino acid withdrawal. Altogether, these results indicate that it is the pool of Rag colocalized with Ragulator (and, ultimately, with Rheb) that is subject to amino acid regulation of raptor binding and, thus, relevant to the ability of the Rag heterodimer to mediate amino acid activation of mTORC1.

**FIGURE 8.**
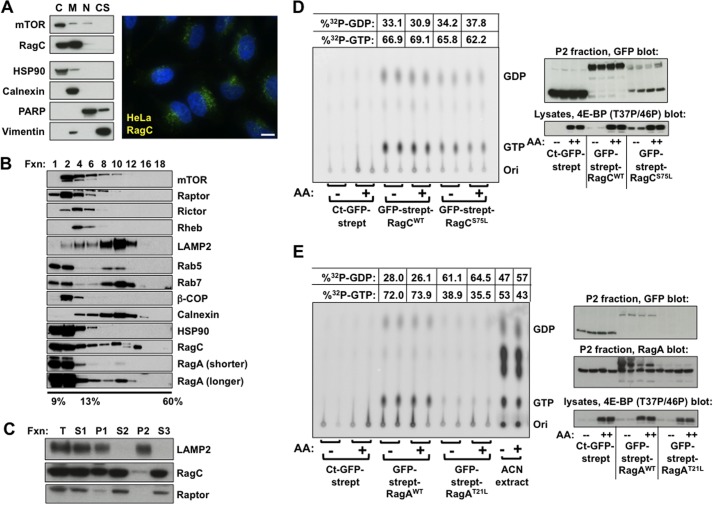
**The effect of amino acid withdrawal on [^32^P]guanyl nucleotide content of recombinant Rag heterodimeric complexes associated with lysosomal membranes in ^32^P_i_-labeled HEK293T cells stably expressing recombinant RagA or RagC variants.** *A*, subcellular distribution of endogenous RagC in HEK293E cells. A commercial kit was employed to extract selected subcellular fractions from HEK293E cells. Markers included HSP90 (cytosol, *C*), calnexin (endoplasmic reticulum and membranes, M), poly-ADP ribose polymerase (*PARP*) (nucleus, *N*), and vimentin (intermediate filaments and cytoskeleton, *CS*). The image shows the immunocytochemical localization of endogenous RagC in HeLa cells. *Scale bar* = 10 μm. RagC, *green*; DAPI, *blue. B*, sucrose density gradient fractionation of HEK293T cells. HEK293T cells were sheared open and fractionated upon a 10-ml 13–60% sucrose density gradient at 100,000 × *g* for 4.5 h. 500-μl fractions were collected, and 50 μl of each even-numbered fraction (*Fxn*), plus the top fraction (1), was subjected to SDS-PAGE and analyzed by immunoblotting as indicated. Two exposures are shown for RagA. *C*, isolation of lysosomes from HeLa cells by differential centrifugation. *T*, cell homogenate; *S1*, supernatant from centrifugation of T at 1000 × *g* for10 min; *P1*, pellet from centrifugation of T at 1000 × *g* for10 min; *S2*, supernatant from centrifugation of S1 at 16,100 × *g* for 30 min; *P2*, the pellet from the latter centrifugation; *S3*, the supernatant after centrifugation of S2 at 10^5^ × g for 30 min. Each fraction was brought to the volume of the original homogenate, and an equal volume was subjected to SDS-PAGE and analyzed by immunoblotting as indicated. *D*, the effect of amino acid withdrawal on the [^32^P]guanyl nucleotide content of stably expressed RagC^WT^ and RagC^S75L^ heterodimeric complexes associated with a LAMP2-containing fraction of ^32^Pi-labeled HEK293T cells. Replicate plates containing the GFP-streptag-RagC variants indicated were incubated with ^32^P in P_i_-free DMEM for 4 h, rinsed, and incubated in either homemade P_i_-free medium (*AA*+) or P_i_-free medium lacking amino acids (*AA*-), each containing ^32^P_i_ as in Fig. 6. The cells were extracted two h later, the fraction corresponding to P2 (*C*) was isolated, and the [^32^P]guanyl nucleotide content of Strep-Tactin-isolated Rag complexes in P2 was determined as in Fig. 6. Immunoblot analyses of GFP in the P2 fraction and 4E-BP(T37P/T46P) in the lysate are shown in the *right panel. E*, the effect of amino acid withdrawal on the [^32^P]guanyl nucleotide content of stably expressed RagA^WT^ and RagA^T21L^ heterodimeric complexes associated with a LAMP2-containing fraction of ^32^Pi-labeled HEK293T cells. The cells and extracts were processed as described in *D*. Immunoblots of GFP and RagA in the P2 fraction and 4E-BPT37P/T46P) in the lysates are shown in the *right panel. Ori*, origin; *Ct*, C-terminal.

In view of these considerations, we sought to determine the effects of amino acid sufficiency on the guanyl nucleotide charging of Rag heterodimers associated with lysosomes. Extraction of HEK293E cells using a commercial kit suggested that ∼50% of endogenous Rag is extracted with cytosolic proteins, the remainder remaining membrane-associated (Fig. 8*A*, *left panel*). Immunocytochemical detection using several polyclonal antibodies against the RagC amino terminus reveals a pattern of speckled, particulate staining within the cytoplasm (Fig. 8*A*, *right panel*). Thus, approximately half of cellular RagC is associated with cytoplasmic membranes. However, the dynamics of Rag membrane localization are not addressed. Differential and sucrose density gradient centrifugation were utilized to further define the subcellular distribution of the Rags, raptor, late endosomal (Rab7), and lysosomal markers (LAMP2) and Rheb (Fig. 8*B*). Homogenates prepared from amino acid-replete and amino acid-deprived HEK293T cells by gentle shear though a fine needle were subjected to sucrose density gradient centrifugation. Amino acid withdrawal did not alter the distribution of raptor or the membrane markers (not shown), indicating that this method is not useful for the evaluation of the amino acid-induced relocalization of mTORC1 to the lysosomal compartment. The vast majority of RagA and RagC cosediments with cytoplasmic and low-density membrane markers (*e.g.* β-COP), and only minor amounts cosediment with the lysosomal marker LAMP2 (Fig. 8*B*). Using differential centrifugation, we generated a membrane fraction (*P2*) that contained all extracted LAMP2 and ∼5–10% of endogenous Rag (Fig. 8*C*). We applied this procedure to HEK293T cells stably expressing recombinant GFP-streptag, GFP-streptag-RagC^WT^, and GFP-streptag-RagC^S75L^ labeled with ^32^P_i_ as above, and examined Rag GTP charging in response to amino acid withdrawal in this LAMP2-enriched fraction (Fig. 8*D*). As observed with total cellular Rag, the fractional GTP charging of lysosome-associated recombinant RagC^WT^ and RagC^S75L^ was ∼70% and was unaffected by amino acid withdrawal.

In contrast to the robust expression of stably expressed GFP-strep fusions of RagC^WT^, RagC^S75L^, and RagA^WT^, the abundance of stably expressed GFP-streptag-RagA^T21L^ in HeLa or HEK293T cells was very low, below that sufficient to cause an inhibition of mTORC1 signaling, as seen during transient expression of this RagA variant. Nevertheless, a comparison of the [^32^P]guanyl nucleotide content of GFP-streptag-RagA^WT^ with GFP-streptag-RagA^T21L^ associated with the lysosomal fraction was accomplished in one experiment (Fig. 8*E*). As with Rag heterodimers containing recombinant RagC^WT^, the [^32^P]guanyl nucleotide bound to heterodimers containing recombinant RagA^WT^ is ∼70% [^32^P]GTP in the presence and absence of extracellular amino acids. In contrast, only ∼35–40% of the [^32^P]guanyl nucleotide bound to Rag heterodimers containing RagA^T21L^ is [^32^P]GTP. Inasmuch as RagA^T21L^ does not bind guanyl nucleotide *in vitro*, the [^32^P]guanyl nucleotide retrieved with these heterodimers is that bound to the endogenous RagC/D partners (the relative abundance of RagC *versus* RagD in the RagA^T21L^ heterodimers is not known). The finding that RagC/D is preferentially occupied by [^32^P]GDP indicates that, in contrast to the lack of detectable GTPase activity in short-term incubations *in vitro*, RagC/D in cells is capable of GTP hydrolysis, whether intrinsic or GAP-mediated. This result should be compared with the finding of Jeong *et al.* (25), who observed that in the x-ray crystal structure of a recombinant wild-type Gtr1-Gtr2 complex isolated from *Escherichia coli*, Gtr1 (equivalent to RagA/B) contained only GTP, whereas Gtr2 (equivalent to RagC/D) contained only GDP (however, at ∼40% the occupancy as compared with Gtr1).

## DISCUSSION

These studies provide two unexpected findings that will require revision of hypotheses concerning the mechanisms by which amino acids regulate Rag function and the manner in which Rags contribute to mTORC1 activation. First, although we confirm that amino acid sufficiency promotes the association of raptor/mTORC1 with recombinant Rag, our results demonstrate that amino acid sufficiency does not regulate the extent of guanyl nucleotide charging of the Rag heterodimer so that amino acids must control the Rag-raptor association through another mechanism. We show that recombinant RagC (wild-type or S75L-containing) heterodimers, whether retrieved from the whole cell or from a LAMP2-enriched membrane fraction, recombinant RagA wild-type heterodimers recovered from a LAMP2-enriched membrane fraction, and endogenous RagA(mostly)-B/C wild-type heterodimers retrieved from whole cell lysates all exhibit a very high fractional content of GTP in both amino acid-replete and amino acid-deprived cells, roughly 60–70%, a fraction similar to that observed for overall cellular guanyl nucleotides. Moreover, withdrawal of amino acids, although strongly inhibitory to mTORC1 signaling and to the binding of endogenous raptor to recombinant Rag heterodimer, does not alter Rag fractional GTP content. Inasmuch as RagC^S75L^ does not bind guanyl nucleotides, these results indicate that the endogenous RagA (mostly) partner is constitutively bound to GTP in the presence or absence of extracellular amino acids. The high level of GTP charging of RagA/B in cells is consistent with the very rapid rate of spontaneous guanyl nucleotide exchange exhibited by the purified Rag heterodimers *in vitro* (Fig. 2, *C* and *D*). Consequently, as compared with the localizing function of Ragulator/LAMTOR, its Rag guanyl nucleotide exchange factor activity detected *in vitro* (18) may not be consequential in cells, at least in regard to the regulation of Rag function by amino acids. On the basis of the high, amino acid-insensitive GTP charging of RagA/B (Figs. 6–8) and the lack of biochemically detectable RagA/B GAP activity in cell extracts regardless of prior amino acid sufficiency (Fig. 3, *D* and *E*), we conclude that the RagA/B GAP activity reported for the cytoplasmic GATOR complex (26) is not activated by amino acid withdrawal. Constitutive charging with GTP and a lack of intrinsic and GAP-stimulated GTPase activity is not unprecedented. The Rho GTPase subfamily of Rnd1, Rnd2, and Rnd3/RhoE exhibits constitutively high charging with GTP. Rnd activity is regulated at a transcriptional level and by phosphorylation (27–29). In contrast to RagA/B, however, RhoE contains substitutions at residues equivalent to Ras12, 59, and 61 that would inhibit Ras GTPase activity. Thus, a rationalization for the low GTPase of the RagA/B is lacking.

The RagA^T21L^-RagC/D heterodimer, wherein bound nucleotide is attributable entirely to the RagC/D partner, exhibits 35–40% occupancy by GTP. This much lower GTP charging is not due to a greater affinity of RagC/D for GDP over GTP inasmuch as GTP and GDP exhibit a similar ability to displace bound guanyl nucleotide from the RagB^T54L^/RagC^WT^ heterodimer (Fig. 2*D*, *bottom panel*). Rather, the lower GTP content of RagC/D is probably due to RagC/D GTPase, either a low level intrinsic activity that is not readily detected *in vitro* or the action of a RagC/D-specific GAP in cells. The Rag GAP of the GATOR complex is reported to be highly selective for RagA/B *in vitro* (26). However, Tsun *et al.* (30) recently identified a RagC/D-specific GAP activity associated with the Birt-Hoge-Dube-associated protein folliculin in complex with folliculin interacting protein 1. In addition, Han *et al.* (31) described a leucine-stimulated GAP activity associated with leucyl tRNA synthetase that is highly preferential for RagD over RagC. Leucyl tRNA synthetase is, therefore, also a candidate RagD GAP, pending determination of the relative RagC/RagD content of the RagA^T21L^ heterodimers, although the unaltered guanyl nucleotide charging of RagC/D upon amino acid withdrawal is inconsistent.

If endogenous RagA/B is ∼70% GTP-charged and the RagC/D heterodimer partner is 35–40% GTP-charged, it might be expected that a wild-type RagA/B-RagC/D heterodimer would be ∼50–55% GTP-charged. However, our estimates for the recombinant GFP-streptag-Rag wild-type heterodimers are consistently in the 60–70% range. One potential explanation for this discrepancy is suggested by the crystal structure of the recombinant Gtr1-Gtr2 heterodimer isolated from *E. coli* (25). Although only GTP is bound to Gtr1 and only GDP is bound to Gtr2, the occupancy of Gtr2 by GDP was ∼40% lower than that of Gtr1 by GTP. They found that Mg^2+^ participates in guanyl nucleotide binding to Gtr1 but not to Gtr2 and speculated that Gtr2 may have a lower affinity for guanyl nucleotide than Gtr1 and may lose GDP during isolation. A preferential loss of [^32^P]GDP from RagC/D during isolation would result in a spuriously high estimate for the [^32^P]GTP content of the wild-type heterodimer.

It is important to emphasize that the high level of RagA/B GTP charging observed is not an artifact of RagA/B charging post-extraction. Although cells are extracted in the presence of Mg^2+^, there is high dilution of cellular guanyl nucleotides. More importantly, during the incubation of the extract with Strep-Tactin (for isolation of recombinant Rag) or the anti RagC antibody (for isolation of endogenous Rag), the [^32^P]GTP in the extract is extensively hydrolyzed, reduced from ∼60 to ∼15% of [^32^P]GDP+GTP (Fig. 6*C*), whereas the Rag-bound nucleotide retrieved from that incubation is ∼70% [^32^P]GTP. Thus, although nucleotides may be dissociating from the RagC/D partner during purification, nucleotide exchange on RagA/B does not occur under the conditions prevailing in these extracts.

A potential caveat to our conclusion that amino acids regulate the Rag-raptor interaction independently of Rag GTP charging is that it is claimed that amino acid regulation of the raptor-Rag interaction occurs at the cytoplasmic surface of the lysosome (17, 18), and most of our experiments examine the guanyl nucleotide charging of Rag isolated from the whole cell lysate. By cell fractionation and immunocytochemistry, we find that whereas approximately half of endogenous Rag is membrane-associated, differential and sucrose density gradient centrifugation indicate that no more than 5–10% of endogenous or recombinant Rag is recovered with LAMP2-containing, *i.e.* lysosomal, membranes. If that is so, then only the guanyl nucleotide charging of that small fraction of total Rag may be relevant to mTORC1 regulation. To address this concern, we isolated the fraction of recombinant Rag retained in the lysosomal fraction and found that guanyl nucleotide charging of this fraction of recombinant Rag was no different than that observed for total endogenous and recombinant Rag.

A lysosomal site for amino acid regulation of mTORC1 is attractive because the generation of amino acids from autophagy occurs at this site, and independent evidence indicates that the V-ATPase proton pump participates physically in amino acid activation of mTORC1 (32, 33). The finding that less than 10% of endogenous Rag colocalizes with lysosomes was surprising. Although loss of Rag from this fraction could have occurred during cell disruption and fractionation, it is clear that Rag that remains lysosomally associated, like the bulk of cellular Rag, exhibits high, amino acid-independent GTP charging. Components of the LAMTOR complex, including p18/pdro, p14, and MP1, as well as V-ATPase assemblies, are distributed widely in the late endosomal compartment in addition to the lysosome, and late endosomes may also participate amino acid regulation of mTORC1 (34). Amino acid sufficiency is reported to promote the binding of raptor to Rag without altering Rag localization or the Rag-LAMTOR association (12, 17). The critical function of Rag in mTORC1 regulation is proposed to be the amino acid-induced colocalization of mTORC1 with Rheb-GTP. However, although Rheb has been found in the Golgi and endoplasmic reticulum (35, 36) and, more recently, with peroxisomes (37) and the mitochondrial outer membrane (38), there is little evidence (39) that Rheb resides in the late endosomal/lysosomal compartment where Rag is bound to LAMTOR. Thus, the steps by which amino acids acting upstream of Rag bring mTORC1 into proximity with active Rheb remain to be fully determined.

A second significant finding of this work relates to the differential effects of Rag mutations on mTORC1 binding and activation. Thus, the RagC^S75L^ mutation, partnered with RagB^WT^, exhibits much stronger binding of raptor than the RagB^WT^/RagC^WT^ heterodimer, but neither Rag heterodimer gives robust mTORC1 activation when overexpressed in amino acid-deprived cells, indicating that raptor binding to a Rag heterodimer is not sufficient for mTORC1 activation. This view is further supported by the finding that, with sufficient overexpression of wild-type RagC (and the accompanying increase in endogenous RagA abundance), raptor associates with Rag heterodimers in a constitutive, amino acid-independent manner without alteration of mTORC1 activity either in the presence or absence of amino acids (Fig. 5). Thus, the raptor-Rag association is necessary, but not sufficient, for mTORC activation. An additional amino acid-regulated step is required that results in the effective approximation of mTORC1 with Rheb-GTP (12, 17), its proximate activator. This putative amino acid-dependent step does not involve altered Rheb (9, 11) or Rag GTP charging (Fig. 6–8) but may involve an alteration in Rag configuration. RagB^Q99L^/RagC^WT^ and RagB^WT^/RagC^WT^ bind raptor comparably *in vitro* and during transient expression (Fig. 1, *A* and *B*), whereas only the former gives strong mTORC1 activation (*C*). Thus, the heterodimer configuration engendered by the RagC/D P-loop mutant partner favors raptor binding but enables little mTOR activation, which depends more strongly on the configuration of the switch I and switch II segments of GTPase domain of the RagA/B partner. There is as yet no information on the structural changes caused by the mutations that render the Rag heterodimer optimally active in mTORC1 regulation. Two reports describe the crystal structure of a Gtr1-Gtr2 heterodimer (20, 25). Both reports describe a U-shaped dimer, with dimerization mediated by the extended C termini and the GTPase domains on the upper limbs of the U. If both Gtr subunits are charged with GTP, their switch regions face in opposite directions, perpendicular to the plane of the dimer with little contact. In contrast, the Gtr1-Gtr2 crystals containing Gtr2 charged with GDP (25) show little change in Gtr1-GTP structure, but the Gtr2 GTPase domain shows an ∼28° rotation from its GTP structure, involving the switch I through switch II segments and resulting in a greatly increased contact with the Gtr1 GTPase domain as well as with its own C-terminal domain. How this marked, GDP/GTP-regulated change in Gtr2 structure affects raptor binding and the location of the Rag residues crucial to raptor binding and how mutations such as RagA^Q66L^ and RagC^S75L^ alter Rag structure remain to be determined. Although it has been assumed that amino acids promote the optimal Rag configuration by altering guanyl nucleotide content, these data point to the likely operation of other mechanisms, *e.g.* amino acid-dependent changes in Rag covalent modification or altered Rag protein-protein interactions.

In conclusion, we find that, in cells, the RagA/B partner of Rag heterodimers exhibits 60–70% fractional GTP charging comparable with the overall cellular GTP/GTP+GDP, whereas 35–40% of the guanyl nucleotide bound to the RagC/D partner is GTP. Most significantly, withdrawal of extracellular amino acids, although capable of inhibiting the Rag-raptor interaction and mTORC1 signaling, does not alter Rag guanyl nucleotide charging. Consequently, the ability of amino acids to promote the binding of mTORC1 to the Rag heterodimer must require some other alteration of Rag, mTORC1, and/or the participation of other proteins. In addition, mTORC1 binding to the Rag heterodimer is not *per se* sufficient for mTORC1 activation. When bound, the configuration of the GTPase domain of the RagA/B partner plays a critical role that is yet to be defined. Considerable evidence indicates the importance of the Ragulator (17, 18), V-ATPase (32), GATOR/SEAC (26), and FLCN-FNIP1 (30, 40) complexes in the amino acid- and Rag-dependent regulation of mTORC1. The present data indicate, however, that these elements do not act through the amino acid regulation of RagA/B guanyl nucleotide charging.

## Footnotes

2 The abbreviations used are:

mTORC1: mammalian target of rapamycin complex 1
TSC: tuberous sclerosis complex
Ab: antibody
AA: amino acid(s)
DPBS: Dulbecco's phosphate-buffered saline
GAP: GTPase-activating protein
GMP-PNP: guanosine 5′-(β,γ-imido)triphosphate.
